# Supplementary material for: Geometric Morphometrics and Genetic Diversity Analysis of Chalcidoidea (Diglyphus and Pachyneuron) at Various Elevations
Source: Insects. 2024 Jul 3;15(7):497. doi: 10.3390/insects15070497 (PMC11277471; doi:10.3390/insects15070497)
Supplement: Supplementary file 1 [file insects-15-00497-s001.zip › Table S1.pdf]

Table S1 The number specimens and sampling sites of eight species

Genus	Species	Sampling sites	Number
Diglyphus Walker	*D. isaea*	10	42
	D. chabrias †	6	35
	D. sabulosus †	5	29
	D. albiscapus	5	22
	D. crassinervis	4	23
Pachyneuron Walker	*P. aphidis*	9	32
	P. grande	4	46
	P. solitarium	10	30

† Representing a new record species from China.
