# Supplementary material for: Geometric Morphometrics and Genetic Diversity Analysis of Chalcidoidea (Diglyphus and Pachyneuron) at Various Elevations
Source: Insects. 2024 Jul 3;15(7):497. doi: 10.3390/insects15070497 (PMC11277471; doi:10.3390/insects15070497)
Supplement: Supplementary file 1 [file insects-15-00497-s001.zip › Table S2.pdf]

Table S2 Information on sequenced specimens with GenBank accession number of COI

Number	Family	Morphospecies	GenBank accession	Sex
1	Eulophidae	*Diglyphus chabrias*_No.7(1)	PP533062	F
2		*Diglyphus chabrias*_No.7(2)	PP535571	F
3		*Diglyphus chabrias*_No.7(3)	PP536635	F
4		*Diglyphus chabrias*_D15	PP542507	
4		*Diglyphus sabulosus*_No. B29(1)	PP542171	F
5		*Diglyphus sabulosus*_No. B29(2)	PP538809	F
6		*Diglyphus sabulosus*_No. B29(3)	PP538811	F
7		*Diglyphus albiscapus*_No. B14(1)	PP536534	F
8		*Diglyphus albiscapus*_No. B14(2)	PP536548	F
9		*Diglyphus albiscapus*_No. B14(3)	PP536562	F
10		*Diglyphus crassinervis*_No.7(2)	PP537116	F
11		*Diglyphus crassinervis*_No.7(3)	PP537592	F
12		*Diglyphus isaea*_No.3	PP537117	F
13		*Diglyphus isaea*_No. B10	PP537586	M
14		*Diglyphus isaea*_No. B4	PP537617	F
15		*Diglyphus isaea*_No.4	PP537627	F
16		*Diglyphus isaea*_No.5	PP537637	F
17		*Diglyphus isaea*1	AB721361.1	-
18		*Diglyphus isaea*2	ON834722.1	-
19	Pteromalidae	*Pachyneuron solitarium*_No. B51(1)	PP537780	F
20		*Pachyneuron solitarium*_No. B51(2)	PP537903	F
21		*Pachyneuron solitarium*_No. B51(3)	PP537911	F
22		*Pachyneuron solitarium*	KY912696.1	
23		*Pachyneuron grande*_No.11(1)	PP537896	F
24		*Pachyneuron grande*_No.11(2)	PP537910	F
25		*Pachyneuron grande*_No.11(3)	PP537916	F
26		*Pachyneuron aphidis*_No.5(1)	PP537902	F
27		*Pachyneuron aphidis*_No.5(2)		F
28		*Pachyneuron aphidis*_No. Z11	PP542405	F
29		*Pachyneuron aphidis*_No. Z12	PP537946	F
30		*Pachyneuron aphidis*1	LC260606.1	-
31		*Pachyneuron aphidis*2	MF979490.1	-
32		*Pachyneuron aphidis*3	KY845889.1	-

33	Pteromalidae	OUT1	OL538149.1	-
34	Eulophidae	OUT2	KJ846111.1	-
