# Supplementary material for: Geometric Morphometrics and Genetic Diversity Analysis of Chalcidoidea (Diglyphus and Pachyneuron) at Various Elevations
Source: Insects. 2024 Jul 3;15(7):497. doi: 10.3390/insects15070497 (PMC11277471; doi:10.3390/insects15070497)
Supplement: Supplementary file 1 [file insects-15-00497-s001.zip › Table S3.pdf]

Table S3 List of sample sites information for *D. isaea*

No.	Population code	Collecting locality	Longitude and Latitude	Altitude(m)	No. of specimens	Date
1	HM	Hami city	93.379722 42.826944	680	12	27-July-2020
2	ALMA	Awatihan-Altun Mountain Ruoqiang county	89.039444 38.068889	3497	6	17-June-2020
3	ALMW	Wuxikuang-Altun Mountain Ruoqiang county	88.973611 37.975000	3849	3	19-June-2021
4	WQ	Wuqia county	75.544444 39.871389	2129	3	29- June-2022
5	AKT	Aketao county	75.248611 38.917222	2487	4	17- June-2021
6	TSK	Tashkurgan county	74.993889 38.372778	3460	5	21- June-2022
7	BL	Bole city	81.001389 44.650556	2096	11	23- June-2022
