# Supplementary figures and images for: Geometric Morphometrics and Genetic Diversity Analysis of Chalcidoidea (Diglyphus and Pachyneuron) at Various Elevations
Source: Insects. 2024 Jul 3;15(7):497. doi: 10.3390/insects15070497 (PMC11277471; doi:10.3390/insects15070497)

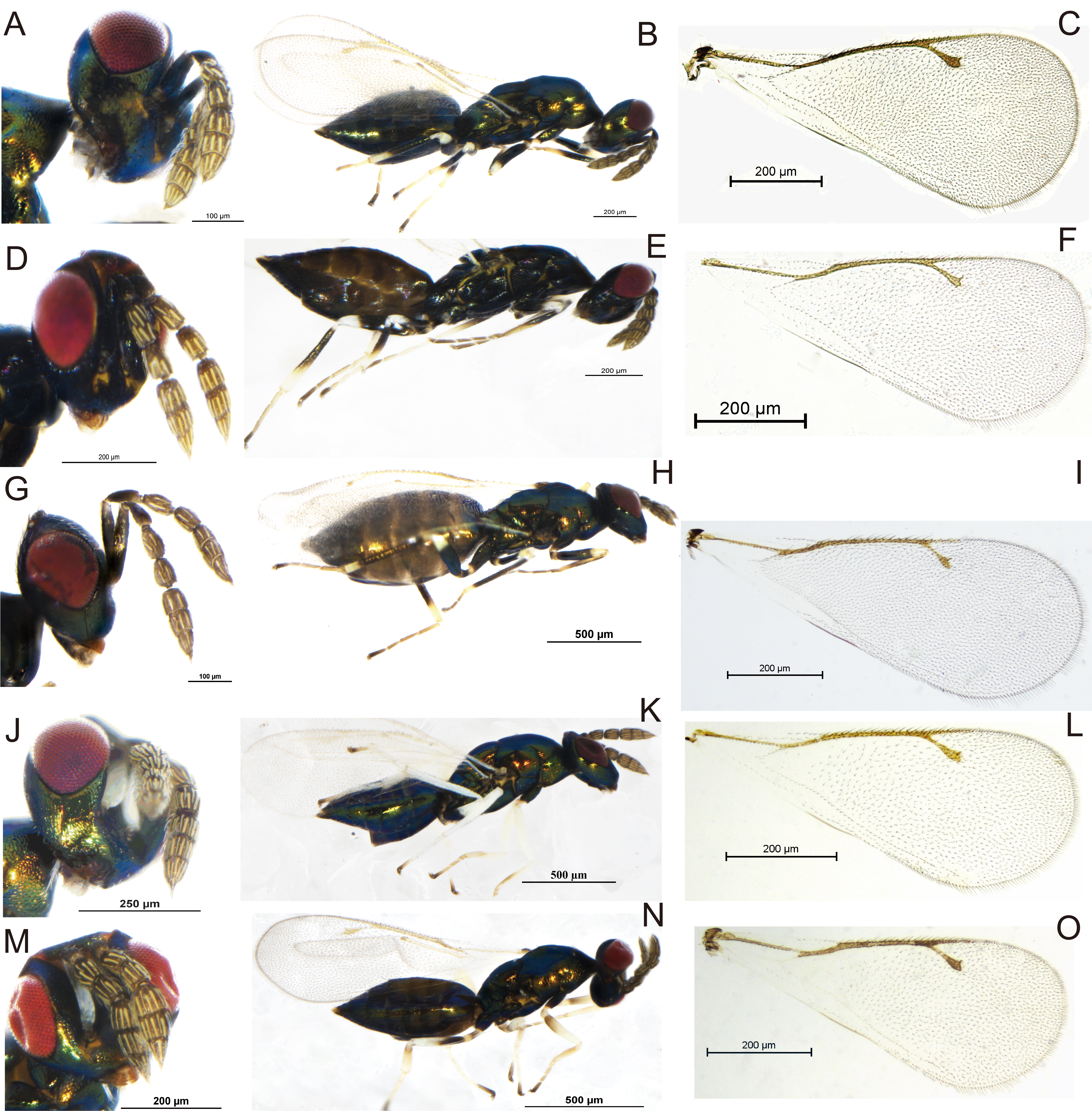

Supplement: Supplementary file 1 [file insects-15-00497-s001.zip › Figure S2.jpg]

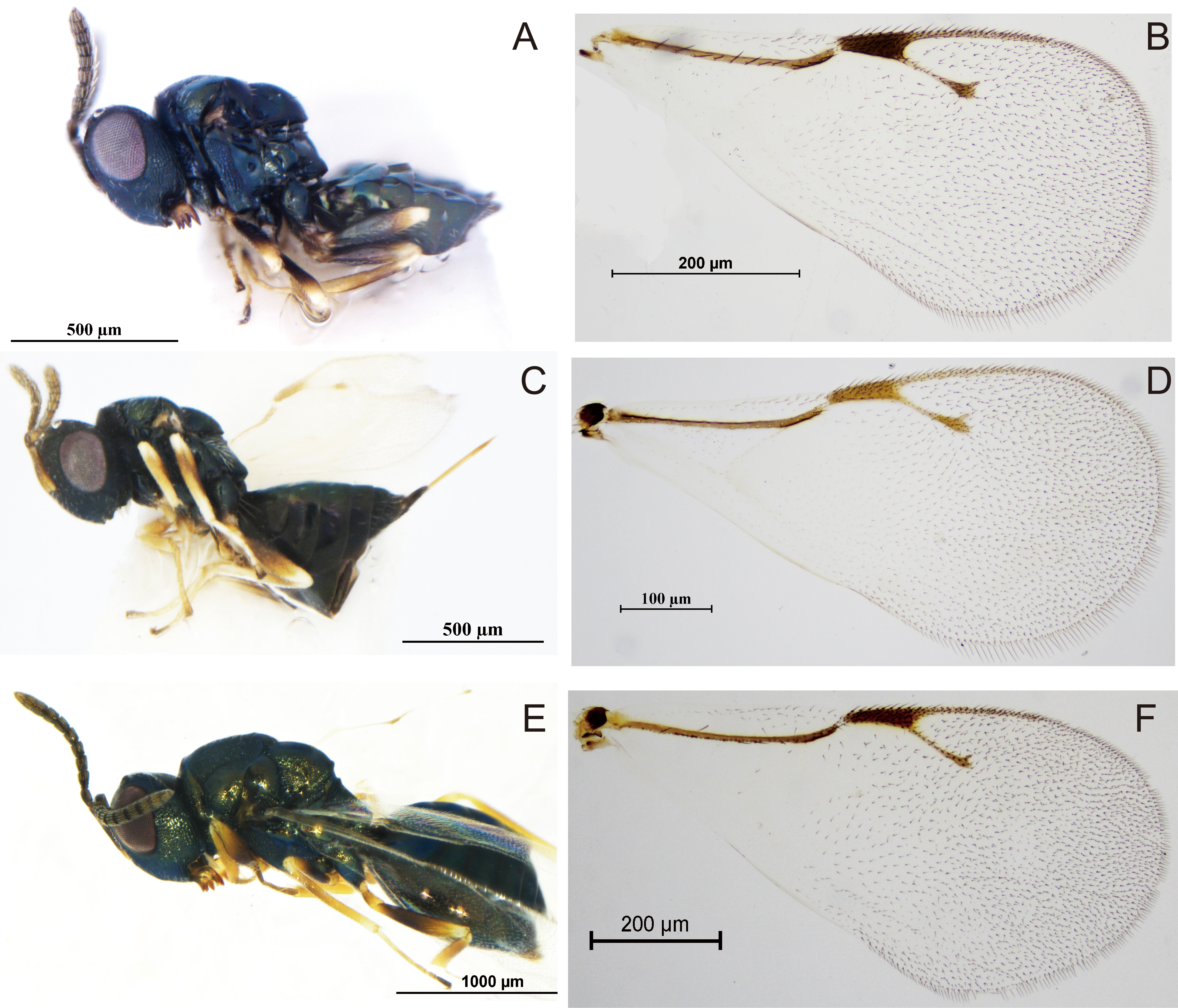

Supplement: Supplementary file 1 [file insects-15-00497-s001.zip › Figure S3.jpg]
